# Achievable resolution for scalable Waveform Digitizing PET readout and comparison with ideal TDC-based readout with an application of DNN to optimal estimation including DOI

**DOI:** 10.1109/NSS/MIC/RTSD57108.2024.10658167

**Published:** 2024

**Authors:** L. Macchiarulo, C. Chock, I. Mostafanezhad, H. Sabet

**Affiliations:** 2 Martinos Center for Biomedical Imaging, Radiology, Massachusetts General Hospital & Harvard Medical School, Boston, MA, USA.; IEEE

**Keywords:** System-on-chip, waveform digitization, picosecond timing, TOF PET, Deep Neural Network

## Abstract

Time Of Flight PET (TOF-PET) is a transformative technology for PET systems, but reaping its fullest potential requires achieving very high spatial and timing resolution, and overcoming dependency on signal variability at the level of the individual pixels. In this study, we try to quantify the advantage of using custom waveform sampling devices versus TDC methodology using realistic estimates of noise and other non-idealities. With the use of DNN methodology we show achievable gain for current and future acquisition systems, and also demonstrate the feasibility of DOI estimation from single side readout. We conclude by arguing for the scalability of such a system based on our experience with compact waveform digitizer design.

## Motivation

TOF measurements currently possible are extraordinarily useful, allowing improved localization of events, reduced statistical noise and higher contrast in diagnostic images. However, currently available readout is hindered by limitations in the readout electronics for robust timing performance in presence of noisy environment, need of very fine accuracy in the threshold definition, necessity of ad-hoc corrections for waveform amplitude (“time-walk”) and shape and the dependency on the scintillator interaction position (Depth of Interaction). We believe that an alternative mechanism consisting in the acquisition of full waveforms (“Waveform Digitization” - WFD) can overcome all these limitations. This work is following our previous modeling effort in [1], and is geared toward the final goal of developing a feasible readout microchip architecture for TOF-PET imaging (**SyMPET**: System on a chip Modular readout for PET) based on Waveform Digitization ASIC technology developed at Nalu Scientific( [2][3]).

## 1. Comparison of TDC and WFD - general considerations

**Figure 1:**
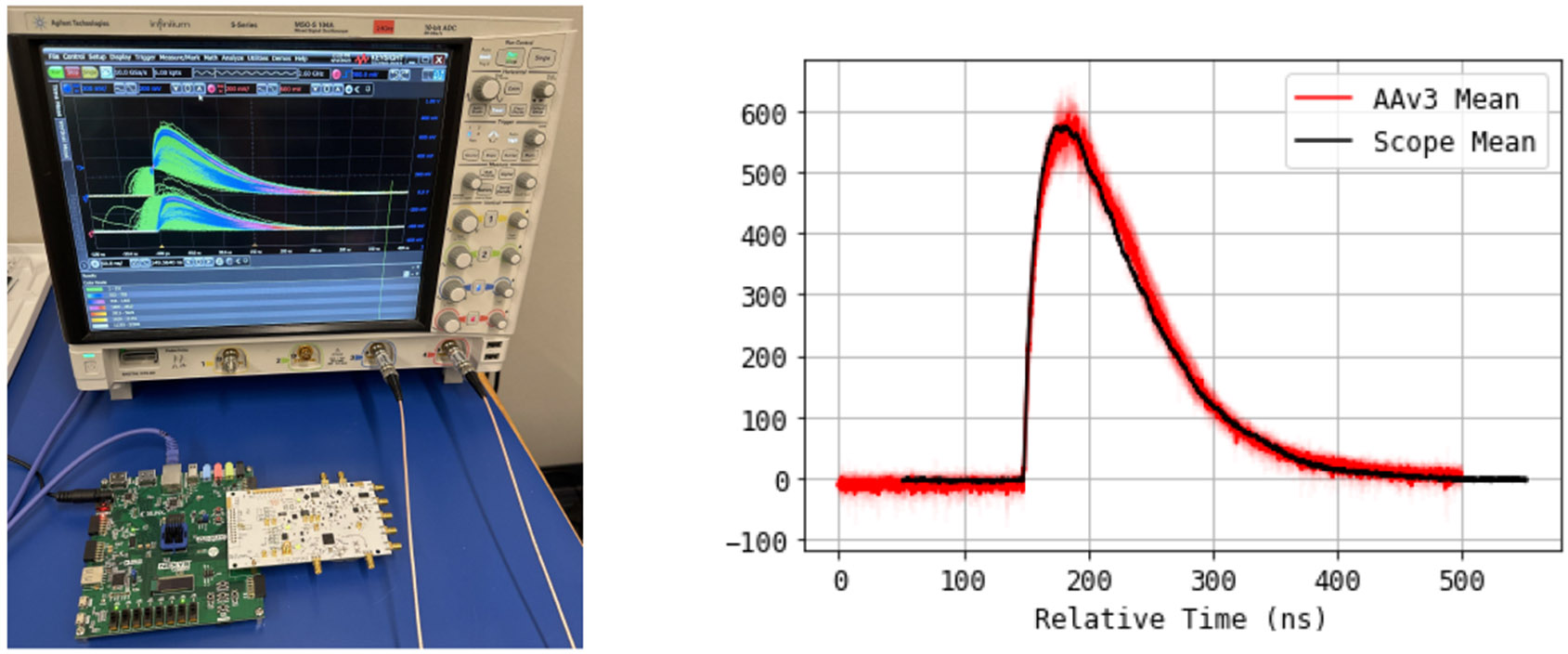
(left) Image of scope in persistent acquisition mode with AARDVARCv3 board below; (middle) Image of Na-22 source and surrounding SiPMs; (right) Average AARDVARCv3 acquisition of light output from a LYSO scintillator through a 2mmx2mm pixel SiPM (red) - compared to an individual waveform from high bandwidth oscilloscope readout.

In [1] we did initially perform an analytic evaluation of the rate requirements of a realistic PET detector and develop Monte Carlo simulations of the full sensor+readout chain to estimate timing resolution, power consumption, channel density, packaging and systems engineering constraints. The details of this modeling effort have been already described in [1] to which we refer for details. The tests for the following simulations were performed through estimation of t0 times for gamma interaction using DETECT, with 1000 events from the same source on a LYSO:Ce crystal model. The analysis of expected waveforms from digitization uses a model for the scintillator and SiPM response that was tuned based on acquisition with a real experimental setup, shown in figure 1. The system uses 2 scintillator crystals, a Na-22 source and 2 Hamamatsu 13660 SiPMs (2mmx2mm) in a black box, read out by a fast sampling scope (10 Gsps) at different bandwidths and with different diode biasing and compared it with a readout using one of our fast sampling chip (AARDVARCv3).

**Figure 2.**
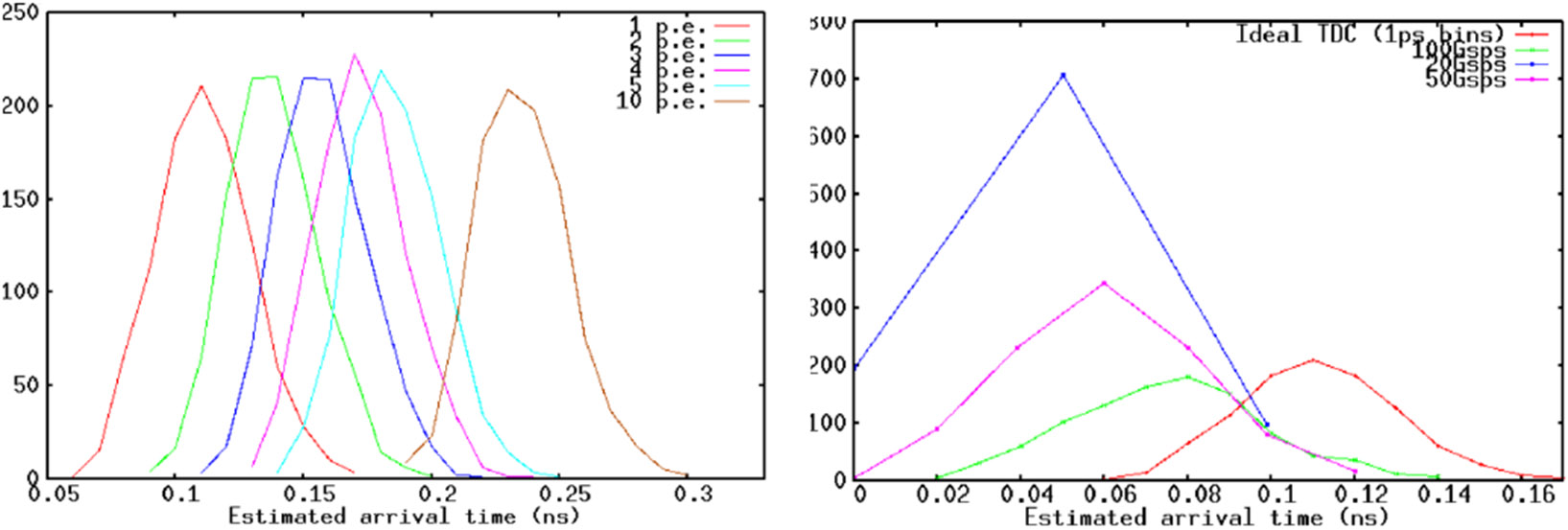
Monte Carlo simulations -left- CTR distributions for 1 ps bin TDC dependent on DC noise- right - CTR distributions for WFD compared with different levels of sampling rates (from 20Gsps (blue) to “ideal” 1 ps binning (red)).

In order to perform a comparison of WFD with traditional TDC method, a series of MC simulations were performed - in these simulations the photon production from a scintillator follows a multi-exponential probability distribution, which was convolved with single photon response extracted from direct measurements of the SiPM used. The parameters of the scintillator response were fitted to the measured response of the scintillator/SiPM combination that was made to match the measured profile after convolution with the single photon response. This gave us a baseline to compare the two approaches with a realistic (and relatively conservative) setup. The derived model was used to generate random waveforms to estimate the resolution of TDC and WFD. Based on the waveforms, an ideal discrimination at a low threshold level was used to simulate traditional TDC, while constant fraction discrimination with sample interpolation was employed for WFD. Clearly, in ideal conditions, such a simple estimation method cannot be performing better than TDC. However, addition of low frequency noise to a TDC distribution or any calibration disturbance that changes the offset between the threshold and the signal causes full degradation - effectively shifting the entire distribution of the estimates. Figure 2, left, shows that, starting from a reference of 1 p.e. threshold distribution, each extra error of 1 p.e. equivalent are additive to the mean, thus effectively degrading the effective time resolution to 95, 162, 217 ps at the 2,3,4 p.e. noise level.

On the other hand, WFC is very robust to low frequency noise or uncalibrated threshold by taking into advantage of its patent knowledge of the baseline before the sensor pulse. Incidentally, high frequency noise effects can also be reduced by the knowledge of multiple samples, though the advantage needs further quantification.

What instead WFD is dependent on is the sampling rate - slower sampling rates obviously correspond to loss in resolution - however, as shown in figure 2, right, the achievable resolution degrades relatively slowly from the ideal (and currently unimplementable case) of 1 ps sampling rate to the reasonable 20Gsps (CTR of 63 ps). In fact, simple discrimination with fast sampling of 10 ps is equivalent to 1 ps TDC binning (CTR of ~50ps) , with these sensor and scintillator parameters.

Besides the robustness that WFD provides compared to TDC, the information of the entire photon distribution is useful for other purposes, for example wave information can correct for the so-called “Time Walk” effect, and more in general for the dependence of the actual arrival time on the effect of the overall SiPM+scintillator variability. This means that the effective crossing point is not entirely indicative of the time of interaction, and time over threshold is a poor estimator of individual component variability. This is particularly true in a realistic system, where a TDC accuracy and binning cannot go down to the single ps level.

## 2. TDC/WFD timing estimation and DNN optimization.

In order to perform a more thorough comparison of WFD with traditional TDC method, another series of MC simulations were performed, using as input the same scintillator and sensor parameters used in the previous section. For these series of simulations, we generated 10,000 random scintillation events, all from the same location in the scintillator, and used different techniques to estimate timing. For the standard TDC, timing was estimated using threshold crossing down to very low voltages (at or below single photon) and ended up with a bast ~40 ps FWHM for the single gamma arrival time (performance degrades with higher thresholds, of course).

In order to derive an unbiased good estimator to use with the case of WFD, the MC waveform traces were subsampled to 20 Gsps and a subset used as a training input for a Deep Neural Network (DNN) - effectively an expedient to optimize the estimation without developing complex algorithms and at the same time an application of Machine Learning on detector space [4].

**Figure 3.**
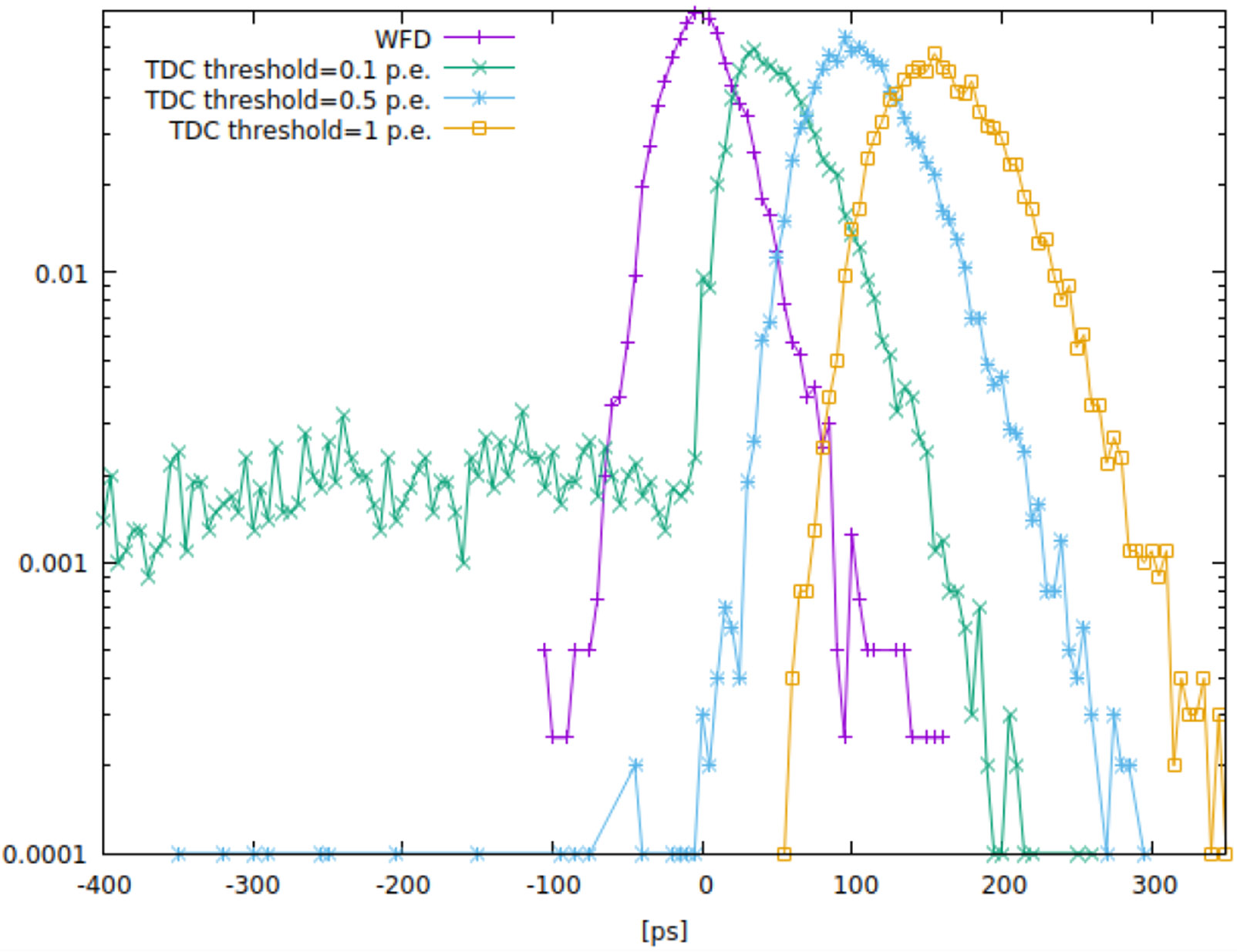
Histograms of residual errors after estimation of gamma arrival time: WFD (purple curve), TDC at different threshold levels. Note the biased nature of the TDC estimate, that moves the mean to the right with the threshold level. X units are in ps.

The predictor achieved a ~50-60ps best result. This is a bit worse than the TDC but the latter assumes perfect binning (down to 1 ps) and the WFD results in an unbiased estimator (i.e. the mean on the error distribution is at 0) contrary to the TDC that is a biased estimator due to the slight walk effect due to small variation between waveforms. In order to estimate the effect of noise, we generated noise with the same spectral response as the SiPM. When a very small amount of noise is added with a frequency spectrum of the SiPM response, (1/10th p.e.) the CRT from the WFD-based DNN estimator remained consistent (~50ps, and unbiased) while TDC degraded to 57ps. However the distribution of the latter (figure 3) presents a long tail that is due to a very high probability of noise triggering. The latter indicates it is not effectively usable in a realistic implementation. In order to partially avoid such noise dependency, a 5x **threshold over the noise (~½ p.e.)** - with a FWHM of 74ps, or fully noise-free triggering at 1 p.e. threshold but with a FWHM of 95ps. Note that this is consistent with the results reported in literature for the best results of TDC ([5]). Note also that the effects which are likely to increase the jitter especially on the TDC case were not incorporated, namely the time-walk effect (all photon responses of the SiPMs are supposed to be identical) and the DOI. We believe that the use of more aggressive parameters for scintillator or SiPM response will give even better results compared to TDC.

## 3. Single-side DOI extraction:

Besides the greatest robustness that WFD provides compared to TDC, the information of the entire photon distribution is useful for other purposes, in particular an estimation of DOI: Such information is not at all extractable from single-sided readouts with TDC mechanism, and TOT provides a very imprecise estimator, while the waveform encodes (via complex pulse-shape patterns), the vertical position of the interaction within the scintillator crystal, which can be used to reduce sources of timing inaccuracy beyond what is normally possible at the level of SiPM readout. In order to estimate the DOI capabilities of the WFD acquisition, a series of waveforms were generated as before, adding the pathlength information to estimate the differential photon arrival times, and the preliminary results via DNN estimations show encouraging discrimination capabilities from single-sided WFD information, with results showing < 1mm resolution.

## 4. Conclusions and future work:

The conclusion of this study is that employing WFD in TOF-PET can provide advantages in resolution, robustness to noise and DOI discrimination. We will pursue more exhaustive evaluations and will use these findings to guide the design of scalable readout asics.
